# A Comprehensive Review of Gestational Diabetes Mellitus: Impacts on Maternal Health, Fetal Development, Childhood Outcomes, and Long-Term Treatment Strategies

**DOI:** 10.7759/cureus.47500

**Published:** 2023-10-23

**Authors:** Vaishnavi S Nakshine, Sangita D Jogdand

**Affiliations:** 1 Medicine, Jawaharlal Nehru Medical College, Datta Meghe Institute of Higher Education and Research, Wardha, IND; 2 Pharmacology and Therapeutics, Jawaharlal Nehru Medical College, Datta Meghe Institute of Higher Education and Research, Wardha, IND

**Keywords:** type 2 diabetes mellitus (dm), blood glucose monitoring, exercise, insulin therapy, pregnancy, obesity, macrosomia, hypoglycemia, hyperglycemia, gestational diabetes mellitus

## Abstract

This review article conducts a comprehensive analysis of gestational diabetes mellitus (GDM) and its ramifications for both maternal health and the well-being of their offspring. GDM is a significant pregnancy complication in which women who have never had diabetes acquire chronic hyperglycemia during their gestational period. In most cases, hyperglycemia is caused by impaired glucose tolerance caused by pancreatic beta cell dysfunction in the background of chronic insulin resistance. Being overweight or obese, having an older mother age, and having a family history of any type of diabetes are all risk factors for developing GDM. GDM consequences include a higher risk of maternal cardiovascular disease (CVD) and type 2 diabetes, as well as macrosomia and delivery difficulties in the newborn. There is also a longer-term risk of obesity, type 2 diabetes, and cardiovascular disease in the infant. Premature birth, hypoglycemia at birth, and shoulder dystocia are also a few of the fetal problems that can result from GDM. Unfortunately, there is no widely acknowledged treatment or preventative strategy for GDM at the moment, except lifestyle modification (diet and exercise) and, on occasion, insulin therapy, which is only of limited value due to the insulin resistance that is commonly present. Although new oral medications for diabetes management, such as glyburide and metformin, show potential, there are ongoing worries regarding their safety over an extended period for both the mother and the child. By identifying gaps in the research, it calls for further investigations and a multidisciplinary approach, ultimately aiming to enhance the management and care for women with GDM, which would impact these affected individuals indubitably.

## Introduction and background

Gestational diabetes mellitus (GDM) is a metabolic condition of pregnancy that presents as newly developing hyperglycemia in pregnant women who did not have diabetes before getting pregnant, and it normally resolves after giving birth [1]. Around 9% of pregnancies around the globe are affected by this prevalent antepartum condition [2]. Although one can develop GDM at any instance during the entire course of pregnancy, it is typically seen between weeks 24 and 28 of pregnancy. Additionally, the prevalence of GDM is growing globally due to an increase in maternal weight gain, maternal age, and inactivity [3]. The etiology of GDM is explained by the maternal pancreas' inability to adjust to the increased insulin demand throughout gestation. During pregnancy, the body becomes less responsive to insulin, which leads to an increased production of insulin by pancreatic beta cells [4]. Insulin, which is secreted by these beta cells, plays a vital role in promoting the uptake of glucose by peripheral tissues, reducing the synthesis of glucose in the liver, and controlling the release of lipids from adipose tissue. However, if regular levels of insulin fail to achieve the desired response from insulin receptors, insulin resistance can develop. Consequently, beta cells must produce more insulin than usual to maintain normal maternal blood glucose levels [1]. This insulin resistance is a natural part of a healthy pregnancy and is induced by placental hormones to ensure the fetus receives the necessary nourishment for proper growth and development. Maternal beta cells respond by increasing their number, insulin production, and release to sustain glucose balance despite insulin resistance [5]. However, when maternal beta cells cannot adapt to the metabolic changes associated with pregnancy, gestational diabetes mellitus (GDM) results in hyperglycemia.

GDM is essential to detect and treat during pregnancy due to the harmful impact it has on both the mother and the fetus, in both the short and long runs. Gestational diabetes can cause short-term pregnancy complications such as high blood pressure, the necessity for a cesarean section (C-section), pre-eclampsia, and difficulty during childbirth [6]. In the long run, it may reappear in subsequent pregnancies, increasing the mother's risk of developing type 2 diabetes later in life [7,8]. Many recent researches have focused on treating GDM with GM-targeting techniques. Several prior research have looked at the influence of probiotics on the progression of GDM, but results have been ambiguous. GDM treatment tries to reduce the hazards for both the mother and the infant by controlling excessive blood sugar levels. Mothers must learn about the illness in order to achieve the best possible blood sugar control in GDM patients. The primary therapies for GDM involve adopting lifestyle changes such as modifying your diet, exercising regularly, and maintaining a healthy weight. Medication may be an option if high blood sugar persists despite all of these changes. GDM medication comprises glucose-lowering drugs, metformin, glibenclamide, and insulin [9]. Women having GDM are recommended to discontinue any medication they were using for the condition postpartum due to the quick return of insulin sensitivity [10].

## Review

Search methodology

**Figure 1:**
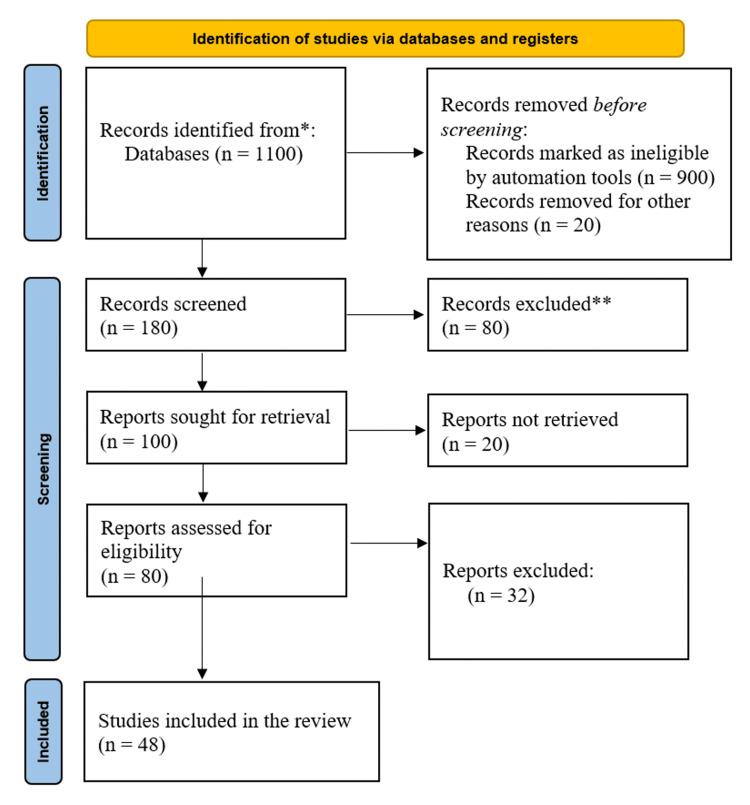
PRISMA flowchart of search strategy PRISMA: Preferred Reporting Items for Systematic Reviews and Meta-Analyses

We undertook a comprehensive search through PubMed and CENTRAL in June 2023 using keywords such as "gestational diabetes mellitus" and "type 2 diabetes mellitus" ((gestational diabetes mellitus [title/abstract]) OR (GDM [title/abstract])) OR (macrosomia [title/abstract]) OR ("gestational diabetes mellitus" [MeSH terms]) AND (("type 2 diabetes mellitus" [title/abstract]) OR (T2DM [title/abstract])) OR ("type 2 diabetes mellitus" [MeSH terms]). Additionally, we looked through the bibliographies of pertinent research to find important references. In July 2023, the search was updated. Two reviewers independently checked the retrieved papers against the inclusion criteria based on the title and abstract first and then the full texts (Figure 1).

Effects on the mother

GDM subsequently causes several short- and long-term complications with regard to maternal health. Along with the challenges of a typical pregnancy, GDM may contribute to depression in prenatal [11]. In many instances, the baby must be delivered surgically due to the increased risk of issues in subsequent pregnancies, such as premature birth and hypertension [12]. Women who have been diagnosed with GDM are significantly more likely to develop diabetes mellitus later in life. Nearly 10% of women with gestational diabetes mellitus are diagnosed with diabetes mellitus shortly post giving birth [13]. Without particular interventions to lower their chance of developing diabetes mellitus, the remainder seem to develop the disease at rates of 20%-60% within 5-10 years following the index pregnancy. However, not all women having gestational diabetes will develop diabetes mellitus, according to limited long-term data from O'Sullivan, but most of them will [14]. Similar to prenatal issues, the risk of postpartum diabetes mellitus is increased by GDM. Regardless, the risk of prenatal problems brought on by GDM is substantially lower than the likelihood of the mother developing diabetes mellitus post-GDM diagnosis. Therefore, it is logical to assume that GDM is a type of prediabetes similar to glucose intolerance in non-pregnant people [15].

**Figure 2:**
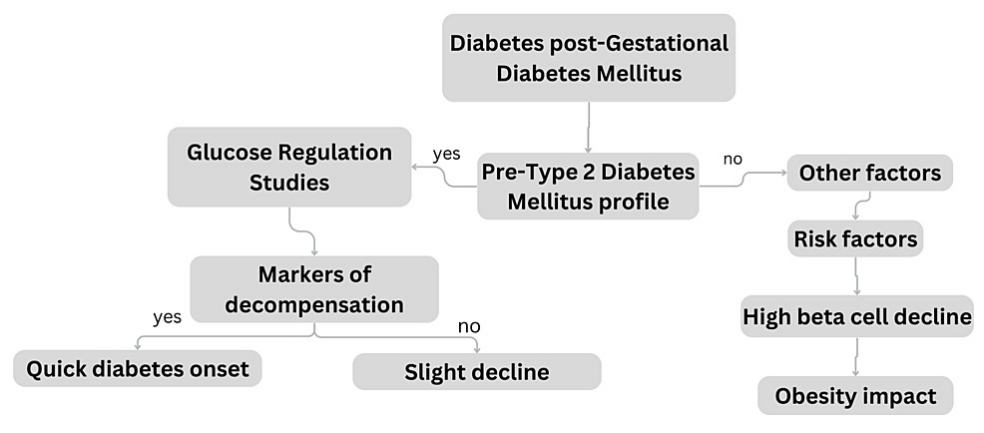
Metabolic factors influencing post-GDM: the role of beta cell function and obesity management GDM: gestational diabetes mellitus Image credits: Vaishnavi Nakshine

Plenty of patients who have diabetes mellitus after GDM meet the pre-type 2 diabetes mellitus (T2DM) profile, as was previously discussed. Studies on the regulation of glucose following GDM over time show declining beta cell remuneration for insulin resistance (chronic) that may also deteriorate as time passes [16]. Markers of rather severe decompensation, such as elevated glucose levels, noticeable insulin resistance, and impaired beta cell activity, are risk factors for the relatively quick onset of diabetes mellitus following childbirth. Women exhibiting these traits might surpass the threshold of glucose levels defining diabetes mellitus following a slight decline in their physical condition [17]. Weight increase, insulin resistance, increasing C-reactive protein levels, and declining adiponectin levels are risk factors for beta cell deterioration at comparatively high rates, which leads to diabetes mellitus [18]. These results imply that the metabolic consequences of obesity serve a key role in the degeneration of beta cells that result in diabetes mellitus. As will be covered below, the most effective defense against the emergence of T2DM after GDM is the amelioration of the detrimental consequences of obesity induced by diet and exercise or by taking medications that improve the biological makeup and operation of adipose tissue [19]. Figure 2 aids in comprehending the multifaceted nature of post-GDM diabetes development and highlights the significance of managing beta cell function and addressing obesity to mitigate this risk effectively.

Metabolic syndrome, which includes obesity and other associated diseases, serves as the foundation upon which T2DM develops. The probability of women with GDM displaying symptoms of metabolic syndrome is higher than it is for women without GDM [17]. A greater frequency of cardiovascular risk factors and cardiovascular events is also linked to previous episodes of GDM [20]. Most mothers who have GDM are obese, and a sizable fraction of obese people also have GDM [21]. According to a meta-analysis, pregnant women who are overweight are 2.14 times more susceptible to be diagnosed with GDM than pregnant women average in weight, obese pregnant women are 3.56 times more likely to do so, and extremely obese pregnant women are 8.56 times more likely [22].

Complications During Pregnancy

Vaginal birth will be more challenging in case the baby is very large. There is a chance of a long labor process during which the fetus could get clung in the birth/vaginal canal, an instrumental delivery may be required (using forceps or a vacuum), or even an unanticipated or emergency cesarean section might be required. A perineal tear (muscle tearing between the vagina and the anus) as well as lacerations and tears of the vaginal tissue are more likely to occur during childbirth than when the infant is of normal size [23]. Moreover, there is a high risk of uterine atony. Heavy bleeding and postpartum hemorrhage may occur as a result of the uterus' muscle failing to contract appropriately. Macrosomic births have an about three- to fivefold increased risk of postpartum hemorrhage and genital tract injury [24]. In addition, if the woman has already undergone a cesarean section, there is an increased probability of tearing of the uterus along the surgical scar tissue from the prior procedure.

Fetal complications and effects

Premature Birth

Preterm delivery is possible as a result of inducing labor early (earlier than 39 weeks and/or early rupturing of the membrane). Although every effort has been made to induce early labor, babies are still at risk for prematurity-related problems, such as breathing and feeding issues, infections, jaundice, admission to a neonatal ICU, and perinatal mortality. Preterm delivery has a prevalence of roughly 10.6% worldwide when combined with several other problematic factors such as obesity and hypertension during pregnancy [25].

Hypoglycemia at Birth

In addition to having a negative impact on mothers, GDM also harms the fetus. The growing fetus can only produce a small amount of glucose; hence, it gets the majority of its glucose from the mother's blood. While maternal insulin does not pass the placenta, maternal glucose does. The modified Pedersen's theory, therefore, states that regardless of glucose stimulation, greater fetal insulin production results from extra glucose transported across the placenta in high and uncontrolled maternal glucose levels [22]. This is corroborated by the placental expression of glucose transport proteins (GLUTs) being found to be higher in pregnancies with insulin-dependent diabetes mellitus [26]. Additionally, insulin is known to have the ability to activate mTOR, a powerful controller of cell proliferation. The placenta's system A and system L amino acid transporters boost cell division and the supply of essential nutrients to the fetus as a result of elevated maternal insulin, which also causes a surge in placental mTOR activity [27]. Maternal hyperglycemia and hyperinsulinemia can result in alterations in the fetus that are comparable to those found in GDM due to the aforementioned causes, which can result in neonatal obesity [28]. An increase in neonatal size at birth, also known as macrosomia, is the result of excessive nutrition storage. The majority of fat is centered in the fetal abdomen and shoulders. Macrosomic babies are born in 15%-45% of GDM pregnancies [22]. Additionally, GDM has been linked to a higher incidence of respiratory distress in newborns [1].

Shoulder Dystocia and Erb's Palsy

Shoulder dystocia, particularly linked to birth trauma, is one of the most serious consequences of administering delivery through the vagina, specifically in macrosomic infants. Newborns weighing 4,500 g or greater are six times more likely than others to experience birth trauma [23], and furthermore, if the birth weight is above 4,500 g, there is an almost 20-fold increased chance of brachial plexus damage [29].

Congenital Anomalies

The most prevalent birth problems include heart defects and disorders of the neural tube, including spina bifida. Congenital abnormalities can result from the growing fetus' organ damage caused by the elevated blood sugar levels of women with GDM [30]. Furthermore, it is not certain if GDM and fetal anomalies are related. Congenital abnormalities are twice as common in women with pre-existing diabetes as they are in non-diabetic individuals, demonstrating a strong association between the two diseases. The data for GDM, however, is inconsistent [1].

Fetal Nutrition

With the onset of GDM, changes in breast milk composition are seen too. Breast milk is a continuously changing fluid with bioactivity that greatly varies from female to female and from phase to phase. Numerous maternal variables, including term and preterm labor, maternal diet, metabolic problems, and diseases [31], have an impact on it. Diabetes mellitus is a long-term metabolic condition that may affect expectant mothers whether it develops before pregnancy or if it develops during pregnancy (a newly formed syndrome) [32]. Citrate, lactose, and total nitrogen levels take 15-24 hours longer for mothers with gestational diabetes to attain levels that are comparable to those of healthy women [33]. Due to the beneficial correlation between mammary gland growth during pregnancy and circulating levels of human placental lactogen, women having gestational diabetes during their pregnancy may have a delay at the beginning of breast milk [34]. Pregnant women affected by gestational DM exhibited elevated levels of cytokines and chemokines in their colostrum. Interleukin (IL)-6, IL-15, and interferon-γ levels were up, whereas IL-1ra and granulocyte-macrophage colony-stimulating factor (GM-CSF) levels were decreased. This led to a modified immune composition of the colostrum [35].

Neonatal complications

Neonatal complications can include delivery trauma, such as shoulder dystocia and a brachial plexus wound, as well as potential hypoxia, hypoglycemia, kernicterus, and jaundice. They may also include bacterial infections and newborn respiratory distress syndrome (NRDS) [2].

Neonatal Jaundice

Prematurity, inadequate nutrition, and increased enterohepatic circulation of bilirubin due to decreased hepatic conjugation of bilirubin are some factors that may contribute to jaundice. Neonates with macrosomia have an elevated oxygen demand, which leads to elevated erythropoiesis and, ultimately, polycythemia [36]. As a consequence of this, as these cells degrade, bilirubin (a by-product of red blood cells) rises, which causes newborn jaundice.

Childhood and adulthood complications

It is generally known that GDM and hyperglycemia in children are related. The research of the Pima Indians in the USA was the first concrete proof that a mother's hyperglycemia may cause her offspring to develop an adult illness. Indeed, children who have diabetic mothers experience an increased risk of obesity, hypertension, and dyslipidemia in later adulthood [37]. In 10 different countries, researchers from the Hyperglycemia and Adverse Pregnancy Outcome (HAPO) study discovered a direct link between maternal hyperglycemia during pregnancy and a rise in hyperglycemia and insulin resistance in children as they grew older [38]. Additionally, compared to offspring of mothers with normal blood sugar levels, GDM progeny had a higher homeostatic model assessment of insulin resistance (HOMA-IR), waist measurement, body mass index (BMI), and triglyceride levels [39]. With about 20% of offspring resulting from GDM having type 2 diabetes and prediabetes by 22, it is plausible that the development of being resistant to insulin raises the chance of the child getting the disease [40]. Along with an increased risk of illnesses including cardiovascular conditions and resistance to insulin, the greater incidence of obesity in children of women with GDM is also linked to an increased risk of other diseases [40]. In addition to hyperglycemia and BMI, children delivered to GDM mothers were shown to have considerably greater cardiovascular risk and adiposity. GDM kids are more likely to experience cardiac arrhythmias and require hospitalization for cardiovascular diseases (CVDs) as a result of increased cardiovascular risk [39]. In addition, GDM offspring are 29% more likely to suffer early-onset cardiovascular conditions such as cardiac failure, high blood pressure, deep vein thrombosis, and pulmonary embolism [41]. All of these researches indicate that the environment in utero affects how metabolic illness is programmed in the child. Population studies have shown that all of these changes experienced throughout childhood are probably to last into adulthood. Numerous studies also indicate that the long-duration impacts of in utero GDM subjection often do not manifest themselves until adolescence, another period that is particularly vulnerable to the development of obesity [23].

Treatment

**Figure 3:**
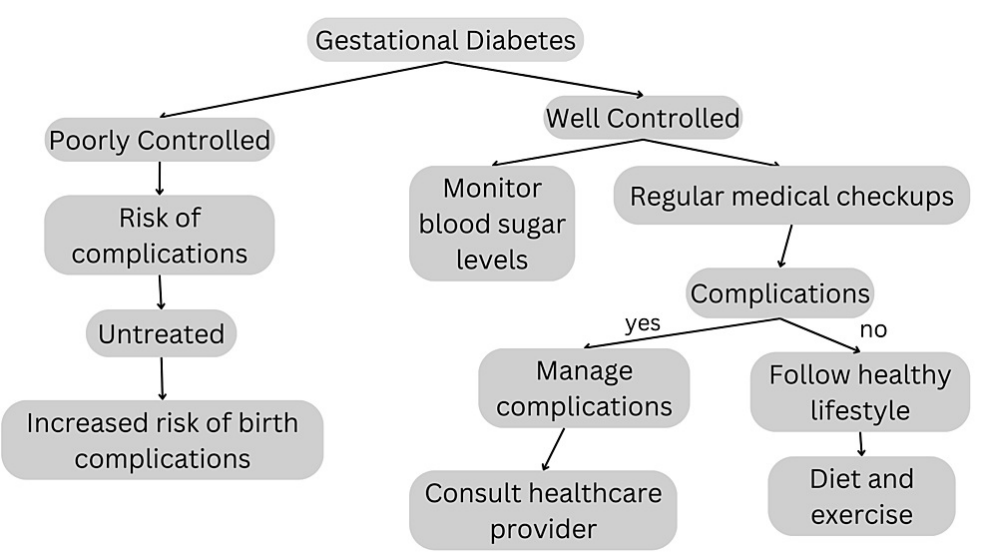
GDM and its control GDM: gestational diabetes mellitus Image credits: Vaishnavi Nakshine

A comprehensive strategy is needed to handle an individual with GDM as best as possible. This entails educating patients on managing pregnancy weight gain, dietary adjustments, nutritional monitoring, and regulating one's blood sugar levels. With enough exercise, dietary changes, and lifestyle adjustments, up to 70%-85% of those with gestational diabetes are curable [42]. For 15%-30% of people, taking medication is necessary. Insulin and oral hypoglycemics are some of them. Figure 3 provides a concise overview of the management strategies to be employed for controlling GDM.

Blood Glucose Monitoring

The majority of organizations advise daily at-home self-glucose monitoring. Presently, daily self-monitoring of postprandial and fasting blood glucose levels is encouraged. The American Diabetes Association (ADA) advises that the target blood sugar levels be 95 mg/dL for fasting and 140 mg/dL or 120 mg/dL for one to two hours, respectively, following a meal. Pre-existing diabetics are the main beneficiaries of pre-prandial glucose monitoring. Screening the levels of hemoglobin A1C is not as helpful for evaluating glucose control in GDM [43].

Dietary Modifications

Some of the dietary strategies mentioned in the literature include the DASH diet (dietary techniques to treat hypertension), calorie-restricted diets, low-glycemic index diets, low-carbohydrate diets, low-unsaturated fat diets, high-fiber diets, and soy-based diets. The emphasis of nutritional advice should be on a balanced diet with reasonable portion sizes, healthy fats, complex carbs, and 20% protein [44].

Physical Exercise

Even in pregnant women with GDM, physical activity and regular exercise have been promoted and are encouraged. The benefits of moderate exercise during pregnancy include a lower risk of gestational diabetes, a lower potential of larger-than-normal newborns, and a lower risk of high blood pressure problems, preterm birth, and fetal growth restriction [45]. Additionally, pregnancy-related lifestyle modifications affect the period of postpartum, reducing the chance of postpartum depression [46].

Pharmacotherapy for GDM Management

**Figure 4:**
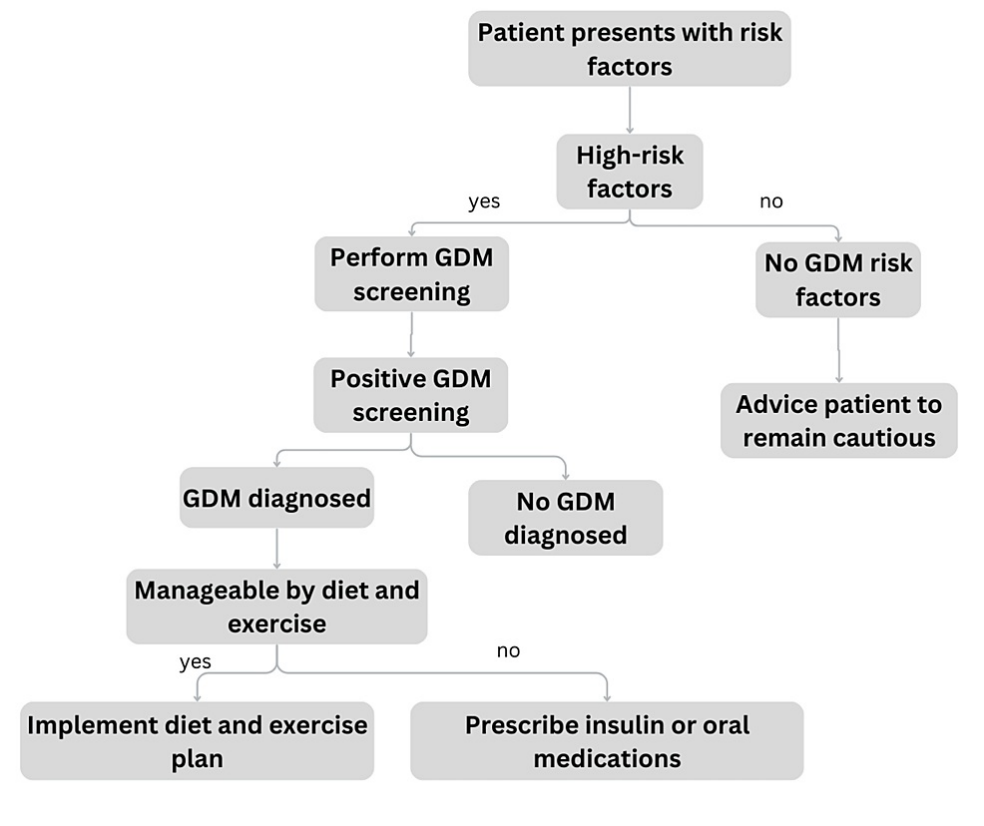
GDM screening and management GDM: gestational diabetes mellitus Image credits: Vaishnavi Nakshine

In about 15%-30% of GDM patients, blood glucose management is insufficient despite suggested dietary and lifestyle changes, necessitating the use of medication [43]. Usually, if hyperglycemia still exists throughout the course of the day after 10-14 days of nutritional and daily living changes, medication courses should be taken into account. Insulin and oral-route medications are administered for patients with gestational diabetes mellitus in order to control hyperglycemia [47]. Insulin provides the most secure outline during pregnancy. The oral medications that have been researched include metformin and sulfonylureas such as glyburide. Large molecules such as insulin cannot pass through the placenta. Metformin and glyburide have been demonstrated to have the capacity to pass the placental barrier and reach the fetus [48]. Figure 4 depicts functions as an illustrative guide outlining the diagnostic and therapeutic procedures for gestational diabetes mellitus.

Table 1 presents an analysis of the traits and features of the articles included in the review.

**Table 1:** Characteristics of the study included in the article GDM: gestational diabetes mellitus, rPL: rat placental lactogen, GCT: glucose challenge test, T2DM: type 2 diabetes mellitus, CVD: cardiovascular disease, LGA: large for gestational age, IDM: infants of diabetic mothers, NIDM: infants of mothers without diabetes

Authors	Year	Findings
Alejandro et al. [1]	2020	This review emphasizes the seriousness of GDM and the problems involved with its care, such as recognizing and dealing with risk factors, accurate diagnosis, and treatment of the condition to avoid associated consequences.
Damm et al. [2]	2016	GDM is becoming more common in most populations, and it is extremely certain that GDM is contributing to the global diabetes epidemic.
Moon et al. [3]	2022	The one-step approach resulted in two to three times the number of women being diagnosed with GDM as the two-step approach.
Lawlor et al. [4]	2010	The findings suggest that maternal glycemia during pregnancy may have a long-term effect on offspring obesity risk.
Parsons et al. [5]	1992	The findings represent the first systematic examination of alterations in islet function throughout pregnancy in rats. Furthermore, they provide evidence that rPL-I may be the essential hormone signal that initiates the fundamental adaptive alterations in islet function associated with pregnancy.
Billionnet et al. [7]	2012	GDM is associated with a significantly elevated risk of unfavorable perinatal outcomes, with insulin-treated GDM having a greater probability of most outcomes than non-insulin-treated GDM.
HAPO Study Cooperative Research Group [8]	2008	Studies show an effective, consistent relationship between reduced maternal glucose levels and increased birth weight and cord blood serum C-peptide levels.
American Diabetes Association [9]	2004	The risk of GDM should be assessed during the first prenatal checkup.
Byrn et al. [11]	2015	According to the findings, depressive symptoms are widespread during the antepartum period, so assessment and education about this illness are critical. A history of depression may also be a warning sign for the onset of GDM.
Tan et al. [12]	2009	Women who tested positive for GCT had a higher risk of having an unfavorable pregnancy outcome. The GCT's role and threshold must be reconsidered.
Buchanan et al. [13]	2012	Even among the vast majority of women who do not have autoimmunity or monogenic diabetes, there is significant variation in GDM.
Metzger et al. [14]	2010	This paper can be used as the basis for internationally recognized guidelines for the diagnosis and classification of diabetes during gestation by diabetes, obstetrics, and other organizations.
Dabelea et al. [15]	2005	The research found that the prevalence of GDM is rising in a multiethnic community that has been universally screened. The growing incidence of GDM shows that the vicious cycle of diabetes in pregnancy initially identified among Pima Indians may be spreading to other ethnic communities in the United States.
Xiang et al. [16]	2010	Women who are the most behind at diagnosis and/or deteriorate the fastest are particularly likely to get type 2 diabetes within 12 years after the index pregnancy.
Retnakaran et al. [17]	2010	GDM and mild glucose intolerance during pregnancy significantly indicate an elevated risk of metabolic syndrome at three months postpartum, lending credence to the idea that women with prenatal dysglycemia may have an underlying latent metabolic syndrome.
Xiang et al. [18]	2010	Weight gain is the main predictor related to deteriorating beta cell compensation for insulin resistance in Hispanic women at high risk of type 2 diabetes, according to these findings. This effect could be mediated by at least two mechanisms: changes in adipokine levels and increased insulin resistance.
Hillier et al. [19]	2007	The findings in a multiethnic US sample imply that hyperglycemia during pregnancy is linked to an increased risk of juvenile obesity.
Retnakaran et al. [20]	2009	Women with diabetes during pregnancy and women who had an oral glucose tolerance test but did not have gestational diabetes had an increased risk of cardiovascular disease after a 12.3-year median follow-up when compared with women who were not given a test for oral glucose tolerance.
Sullivan et al. [21]	2012	Medical therapies that prevent the progression of T2DM, such as metformin, might turn out to be our major line of defense against CVD in women with GDM.
Kamana et al. [22]	2004	This study examines research that investigated the impact of GDM and fetal macrosomia on delivery outcomes, as well as macrosomia-related problems, and provides an assessment of mother and fetal health.
Lazer et al. [24]	1986	Aside from women who have already delivered a macrosomic child, cesarean delivery of the macrosomic fetus is strongly advised.
Ehrenberg et al. [25]	1986	Obesity and pregestational diabetes are both linked to a higher risk of LGA delivery.
Illsley et al. [26]	2020	This review looks at how the localization, function, and evolution of placental glucose transport systems vary during fetal development, as well as the transport and metabolic abnormalities seen in prenatal diseases.
Rosario et al. [27]	2016	Obese mice with activated placental insulin/IGF-I/mTOR and leptin signaling pathways boost placental amino acid transport and contribute to higher fetal growth.
Logan et al. [28]	2017	IDM patients had much more obesity than NIDM patients.
McFarland et al. [29]	1986	Findings show that there is a considerable risk of major birth damage related to instrumental midpelvic delivery.
Eidelman et al. [31]	2012	Breastfeeding and the usage of human milk provide distinct nutritional and non-nutritional benefits to the infant and mother, which improves infant, child, and adult fitness and child milestones.
Sibiak et al. [32]	2020	Diabetes along with obesity, in both human and animal models, can disrupt the physiological production and biological action of placental lactogen.
Al-Biltagi et al. [33]	2021	Maternal diabetes has a major impact on the structure and function of the fetal heart and fetal placental circulation.
Hartmann et al. [34]	2021	Over 80% of women who delivered prematurely and were producing milk for their newborn had a delayed initiation of lactation, defined as one or more lactogenesis II markers in their milk that were more than 3 SD higher than the mean of full-term women on day 5 postpartum.
Bitman et al. [35]	1989	Diabetes causes alterations in lipid metabolism in the mammary gland, which alters the content of the diabetic mother's milk.
Holt et al. [36]	2004	Metformin and glibenclamide drugs may be effective and secure alternatives to insulin for the management of gestational diabetes, with metformin being preferred.
Pettitt et al. [37]	1983	Obesity develops in childhood and early adulthood as a result of the perinatal environment of diabetic women's infants.
Boerschmann et al. [39]	2010	The fact that overweight risk is mostly connected with maternal obesity shows that genetic predisposition plays a role in childhood growth among these children.
Yu et al. [41]	2019	Offspring of diabetic mothers, particularly those with a history of cardiovascular diseases or diabetic complications, had higher rates of early-onset heart diseases from childhood to early adulthood.
Lende et al. [44]	2020	Regular examinations along with testing, at least once per 1-3 years, are indicated for early detection of type 2 diabetes and to prevent long-term complications of diabetes mellitus.
Gregg et al. [45]	2017	Regular exercise throughout pregnancy enhances general wellness and aids in the maintenance of a suitable gestational and fetal weight increase.
Brown et al. [46]	2017	Women who received lifestyle treatments were comparatively less inclined to experience postnatal depression and were more likely to meet their postpartum weight goals.
Landon et al. [47]	2002	A randomized clinical trial of moderate gestational diabetes mellitus determines if early detection and treatment of the condition improve neonatal morbidity.
Lv et al. [48]	2015	Aspart, glargine, and detemir are all safe alternatives for treating diabetes during pregnancy; in this research conducted, these insulin analogs did not increase difficulties for mothers or fetuses.

## Conclusions

Global health continues to be seriously impacted by GDM, the most common metabolic condition during pregnancy. Characterized by elevated blood sugar levels during pregnancy, it demands our attention and a deeper understanding due to its significant impact on the health of expectant mothers and their children. This review shed light on the immediate and long-term consequences of GDM on pregnant women. Short-term consequences encompass a higher likelihood of gestational hypertension, cesarean sections, and other perinatal complications. Long-term implications involve an increased risk of developing type 2 diabetes postpartum, highlighting the importance of continued monitoring and care for women who have experienced GDM. Furthermore, GDM's influence on offspring is a matter of critical concern. This review underscores that children of GDM-affected mothers face a higher risk of developing conditions such as obesity, hypertension, and insulin resistance, which can persist into adulthood. Understanding these intergenerational health implications is vital for proactive prevention and management.

To address GDM effectively, a multifaceted approach is required. This approach includes vigilant blood glucose monitoring, dietary modifications, regular physical activity, and, when necessary, pharmaceutical interventions. The effective management of GDM relies on a collaborative effort between healthcare professionals and expectant mothers, emphasizing education and tailored care. A clinical dietitian should provide dietary advice to all women with GDM, as dietary counseling is the cornerstone of GDM treatment. Particular attention should be paid to carbohydrate intake, as carbohydrate type, amount, and distribution all play a significant role in postprandial blood glucose levels. While considerable progress has been made in GDM research and treatment, there are still gaps in knowledge and variances in clinical recommendations. Moreover, a variety of therapy alternatives for GDM are discussed, although existing data do not support the effectiveness of these approaches over the long term. Future research must focus on a more comprehensive understanding of the long-term cardiometabolic risks that the offspring of GDM-affected mothers may face. In addition, for the prevention and control of GDM, an integrated strategy combining population-wide preventive management, intensive health education, early detection, and multidisciplinary care programs should be strengthened, which could help reduce the risk of GDM and associated complications in the general population and high-risk individuals, improve maternal and neonatal pregnancy outcomes, and promote long-term health.
